# The Mediating Role of Placental Weight Change in the Association Between Prenatal Exposure to Thallium and Birth Weight: A Prospective Birth Cohort Study

**DOI:** 10.3389/fpubh.2021.679406

**Published:** 2021-07-02

**Authors:** He Zhou, Xiaoli Sun, Yiding Wang, Yufeng Ye, Hanwei Chen, Qingsong Chen, Guanhao He, Jiaqi Wang, Xin Liu, Moran Dong, Dengzhou Chen, Guimin Chen, Lixia Yuan, Jianpeng Xiao, Jianxiong Hu, Weilin Zeng, Zuhua Rong, Qianqian Zhang, Mengya Zhou, Lingchuan Guo, Yanyun Lv, Jingjie Fan, Yudong Pu, Wenjun Ma, Bo Zhang, Tao Liu

**Affiliations:** ^1^School of Public Health, Guangdong Pharmaceutical University, Guangzhou, China; ^2^Guangdong Provincial Institute of Public Health, Guangdong Provincial Center for Disease Control and Prevention, Guangzhou, China; ^3^Gynecology Department, Guangdong Women and Children Hospital, Guangzhou, China; ^4^Food Safety and Health Research Center, School of Public Health, Southern Medical University, Guangzhou, China; ^5^Guangzhou Panyu Central Hospital, Guangzhou, China; ^6^School of Public Health, Southern Medical University, Guangzhou, China; ^7^School of Public Health, Sun Yat-sen University, Guangzhou, China; ^8^State Key Laboratory of Environmental Criteria and Risk Assessment, Chinese Research Academy of Environmental Sciences, Beijing, China; ^9^Affiliated Jiangmen Hospital of Sun Yat-sen University, Jiangmen, China; ^10^Department of Prevention and Health Care, Shenzhen Maternity & Child Health Care Hospital, Southern Medical University, Shenzhen, China; ^11^Songshan Lake Central Hospital of Dongguan City, Dongguan, China; ^12^School of Medicine, Jinan University, Guangzhou, China

**Keywords:** thallium, heavy metal, birth weight, placental weight, birth cohort study

## Abstract

**Background:** Previous studies have demonstrated the embryotoxicity and fetotoxicity of thallium (Tl). However, the effects of prenatal exposure to Tl on birth weight and placental weight and the mediating role of placental weight in the association of Tl with birth weight remain unclear.

**Methods:** We recruited 2,748 participants from the ongoing Prenatal Environment and Offspring Health Cohort (PEOH Cohort) study, which was initiated in 2016 in Guangzhou, China. The Tl concentrations in maternal urine samples collected during the first and third trimester were determined by inductively coupled plasma mass spectrometry. Birth weight and placental weight were extracted from maternal medical records.

**Results:** Pregnant women exposed to the highest tertile of Tl in the first trimester (β = −42.7 g, 95% CI: −82.3, −3.1 g) and third trimester (β = −50.6 g, 95% CI: −99.0, −2.3 g) had babies with lower birth weights than those exposed to the lowest tertile. We also found significant negative associations of exposure to Tl concentrations in the first and third trimester with placental weight. Mediation analyses showed that 50.3% (95% CI: 15.9, 79.2%) and 33.5% (95% CI: 1.3, 80.3%) of the effects of Tl exposure in the first and third trimester on birth weight were mediated by decreased placental weight.

**Conclusion:** Our results suggest that prenatal exposure to Tl is negatively associated with birth weight and that this association may be mediated by decreased placental weight.

## Introduction

Birth weight is the indicator that is most commonly used to characterize intrauterine growth and development. Low birth weight (LBW <2,500 g) has become one of the main risk factors for global disease burden (1). Compared with infants with normal birth weight, infants with LBW are at higher risk of mortality, morbidity, impaired cognitive development, neurological and behavioral problems, and chronic diseases in adulthood (2–4). The global prevalence rate of LBW in 2018 was 15.5%, which amounts to ~20 million LBW infants born each year, and 96.5% of these LBW infants were born in developing countries (1). In China, the incidence rate of LBW ranges from 2.5 to 9.4% (5).

Previous studies suggested that low birth weight is associated with socioeconomic factors, lifestyle, genetic factors, and environmental exposure, but the exact causes of fetal reduced birth weight remain unclear (6–8). Recent studies have indicated that exposure to environmental chemicals, including heavy metals, can decrease birth weight (9–11). During pregnancy, fetuses undergo critical processes of the brain and nervous system growth, rapid cell proliferation, immature organ development, and metabolic changes, which makes them susceptible to toxic and harmful substances in the environment (12, 13).

Thallium (Tl) is a natural constituent of the Earth's crust that is present in nearly all environmental media. Tl and its compounds have potential applications in industries, commerce, and manufacturing, for the development of products such as semiconductors, electronic equipment, crystals, and costume jewelry (14). In recent decades, anthropogenic activities, such as mining activities, coal and oil combustion, cement plants, and refining processes, have greatly increased the emissions of Tl into the environment (2, 15). It is estimated that ~5,000 tons of Tl per year are released into the environment, resulting in exposure of the general population, which has raised concerns for human health (16, 17). Researchers have reported that any amount of Tl in the body is abnormal and can disrupt protein synthesis, cell cycle progression, and apoptosis, and energy metabolism (15, 18).

Several studies have investigated the associations of prenatal exposure to Tl with adverse birth outcomes (19–22) and children's health (23–25). In the studies that investigated the associations of Tl exposure with adverse birth outcomes, birth weight was a commonly selected indicator that was used to characterize fetal development. For example, a cross-sectional study observed inverse associations of maternal peripheral blood and cord blood Tl concentrations with birth weight (21). A case-control study conducted by Xia et al. found that higher maternal urinary Tl levels were significantly associated with an increased risk of LBW (22). Nonetheless, at present, prospective evidence regarding the association of Tl exposure with reduced birth weight is still limited, and more research is needed.

Previous studies have provided consistent evidence that the placenta plays a vital role in fetal development (26, 27). The placenta, which is a transient organ during gestation, is involved in the implantation of the early embryo, produces hormones to facilitate fetal growth, and exchanges nutrients, oxygen, and waste products between the developing fetus and the mother. It has been demonstrated that the placenta plays an important role in the effects of environmental contaminants on fetal health (28, 29). For instance, Niu et al. observed that decreased placental weight partially mediated the adverse effects of maternal second-hand smoke on low birth weight (29). Hence, it is plausible that placental weight may also play a mediating role in the association of Tl exposure with birth weight. However, we did not find any studies that investigated the mediating role of placental weight in the effects of Tl on birth weight. Elucidating the role of placental weight could extend our understanding of the effects of heavy metals on fetal health.

In this study, we recruited participants from the ongoing Prenatal Environment and Offspring Health Cohort (PEOH Cohort) study, which was initiated in 2016 in Guangzhou, China. We aimed to investigate the relationship between prenatal Tl exposure and birth weight in a Chinese population and to further illustrate the role of changes in placental weight in the association between prenatal Tl exposure and reduced birth weight.

## Materials and Methods

### Study Population

The PEOH study is a prospective cohort study that was initiated in 2016 in Guangzhou, China. The PEOH study aimed to investigate the adverse effects of prenatal exposure to environmental factors on fetal health and to further illustrate the underlying biological mechanisms. Pregnant women were initially recruited from the antenatal care outpatient department in Guangzhou Panyu Central Hospital (the largest hospital in Panyu district) in Guangdong, southern China, between January 2016 and June 2017. All the participants met the inclusion criteria: (1) gestational weeks from 1 to 13; (2) aged 18–50 years; and (3) no comorbidity with the following diseases: hyperthyroidism, heart disease, chronic kidney disease, tuberculosis, and psychiatric disease. Detailed information has been presented elsewhere (30–35). In this study, we recruited women with singleton pregnancies and without occupational exposure to Tl as study participants.

The present study was approved by the Ethics Committee of Guangdong Provincial Center for Disease Control and Prevention and was registered in the Chinese Clinical Trial Registry (ChiCTR-ROC-17013496). Every recruited participant was provided with a detailed introduction and explanation of this study and signed the informed consent form.

### Baseline and Follow-Up Investigations

Each participant was initially recruited early in pregnancy and was asked to complete a face-to-face interview using a questionnaire including demographic characteristics, lifestyle behaviors, residential addresses and information of address change, household living conditions, work environments, activity patterns, mode of transportation, history of diseases, and diet. The prenatal care records of the participants were extracted from the hospital information system. Besides, a 15.0-mL sample of clean, midstream urine was collected from each participant. All the information was saved as baseline information, and a follow-up profile was established for each participant.

A follow-up investigation was conducted for each participant during their hospitalization for delivery (the third trimester), during which the second questionnaire interview was conducted, and another 15.0-mL of clean, midstream urine was collected. The birth record of each infant was extracted from the maternal medical records. The follow-up investigation was completed in December 2017 (Supplementary Figure 1).

### Endpoints

The information on birth weight and placental weight were derived from maternal medical records. Midwifery nurses measured the birth weights and placental weights within 1 h after delivery on an electronic balance in grams. The placentas were fresh with membranes and umbilical cord attached and with not bled dry.

A total of 4,928 pregnant women were initially recruited in the baseline investigation, and 4,279 were successfully followed up (86.8%). We further excluded participants who did not provide urine samples in the baseline investigation (*N* = 2,053), for whom the birth weight or placental weight was not recorded (*N* = 24), had multiple births (*N* = 31), had stillbirths (*N* = 3), or lacked key variables, such as maternal education (*N* = 1), family income (*N* = 12), and prepregnancy BMI (*N* = 2). As a result, 2,153 pregnant women were included in the analysis to examine the association of Tl exposure during the first trimester with birth weight.

In the 4,279 followed-up participants, we excluded participants who did not provide urine samples (*N* = 2,839) during their hospitalization for delivery, for whom no information on birth weight or placental weight was recorded (*N* = 41), had multiple births (*N* = 9), had stillbirths (*N* = 1), or lacked key variables, such as maternal education (*N* = 1), family income (*N* = 14), and prepregnancy BMI (*N* = 3). A total of 1,371 pregnant women were finally included in the analysis to examine the association between Tl exposure during the third trimester and birth weight. Finally, a total of 2,748 pregnant women who were included in these two subgroups were analyzed in this study. Of these, 1,377 pregnant women provided urine samples only during the first trimester; 595 only during the third trimester and 776 both during the first and the third trimester.

### Determination of the Urinary Concentrations of Tl and Creatinine

All the urine samples were collected and stored in polypropylene tubes at −80°C until analysis. Before measurement, the urine samples were thawed at room temperature until completely melted. The detailed methods for measuring the Tl and creatinine concentrations have been described elsewhere (35). Briefly, the urinary concentration of Tl was quantified by inductively coupled plasma mass spectrometry (ICP-MS) (Agilent 7700x, Agilent Technologies) at the laboratory of Guangdong Provincial Center for Disease Control and Prevention. The ICP-MS operating conditions included an RF power of 1,550 W, plasma gas flow of 15.00 L/min, carrier gas flow of 1.14 L/min, and helium gas flow of 4.5 L/min. The intra-assay coefficient of variation (CV) was 4.44%, whereas the mean of inter-assay CV was 5.19%. The limit of detection (LOD) for Tl was 0.03 μg/L. Samples with a concentration below the LOD were imputed as half of the LOD. Forty-six samples had Tl levels below the LOD in the first trimester, and 104 samples had Tl levels below the LOD in the third trimester. We used urinary creatinine to calibrate the urinary Tl concentration. The concentrations of urinary creatinine were assessed by an automatic biochemical analyzer (Hitachi 7600-020) according to the improved Jaffe reaction to correct the urine volume differences and individual differences.

### Covariates

Confounders were defined as variables such that the regression coefficient between exposure and outcome change by more than 10% when these variables are controlled for (36). Variables that met the criteria for confounding were included in the final analyses: maternal age, maternal education, family income, gestational week, parity, gravidity, infant sex, vegetable consumption, fruit consumption.

### Statistical Analysis

Continuous variables with a normal distribution were expressed as the mean ± standard deviation (SD), and continuous variables with a non-normal distribution were described as the median (25–75th percentile). ANOVA or *t*-tests were used to compare the differences in the distribution of neonatal birth weight among subgroups. The urinary Tl concentrations were corrected by creatinine (CC-Tl). A multiple linear regression model was used to estimate the associations (β, 95% CI) between each interquartile range (IQR) of maternal urinary ln-Tl levels and birth weight. And the model was also used to estimate the associations between the second and third tertiles of ln-Tl concentration (reference: first tertile) and birth weight. The ln-Tl concentrations were estimated in the baseline (the first trimester) and follow-up investigations (the third trimester). The multiple linear regression model was also used to investigate the associations between (a) maternal urinary ln-Tl levels and neonatal birth weight, (b) maternal urinary ln-Tl levels and placental weight, and (c) placental weight and birth weight.

**Figure 1:**
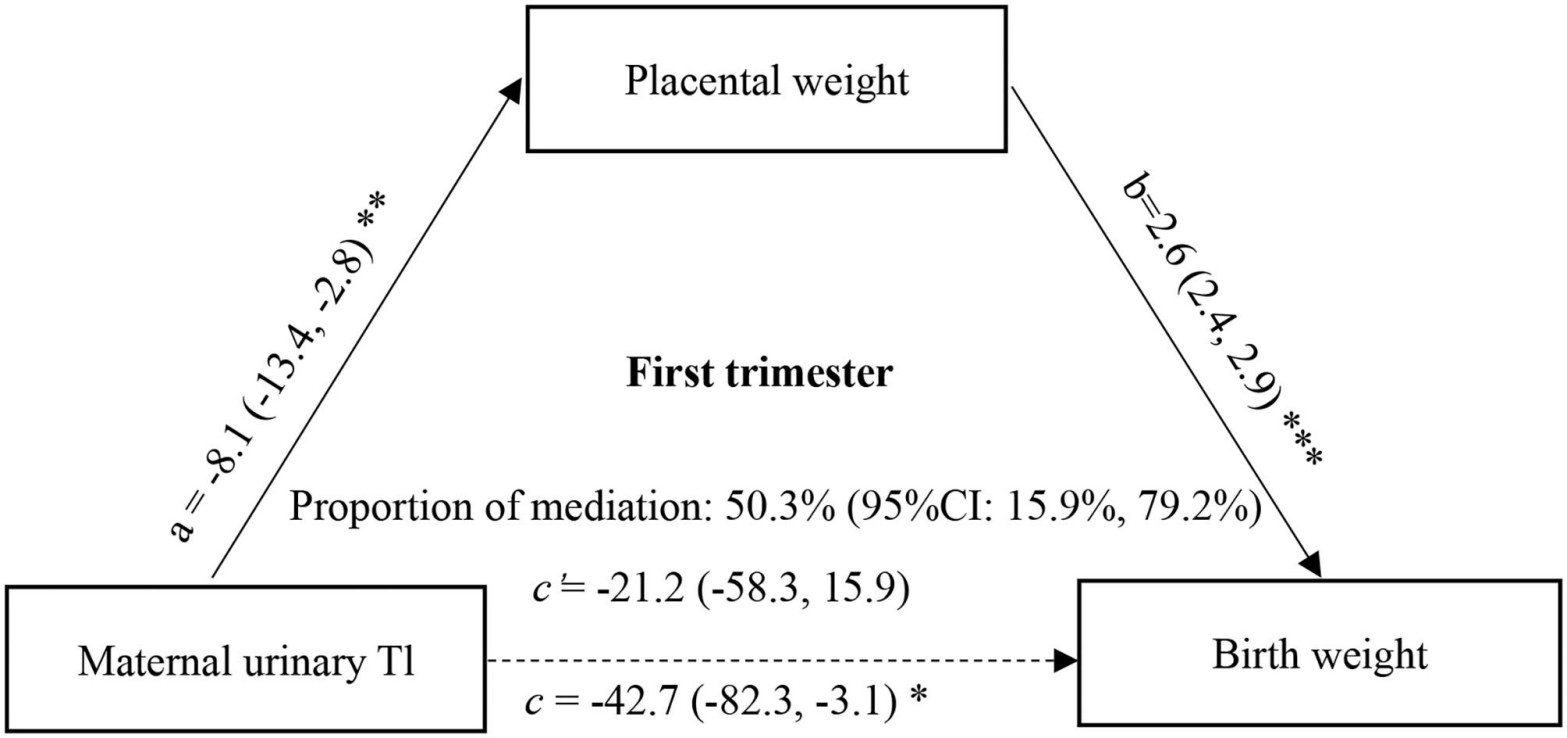
Mediation effect of placental weight on the association of maternal urinary Tl exposure during the first trimester with birth weight. a, c, and c' were estimated for the highest vs. the lowest tertile of ln-Tl exposure, and b was estimated for each unit increase in placental weight. All effect sizes were adjusted for final analyses: maternal age, maternal education, family income, gestational week, parity, gravidity, infant sex, vegetable consumption, fruit consumption. ^***^P < 0.001, ^**^P < 0.01, ^*^P < 0.05.

**Figure 2:**
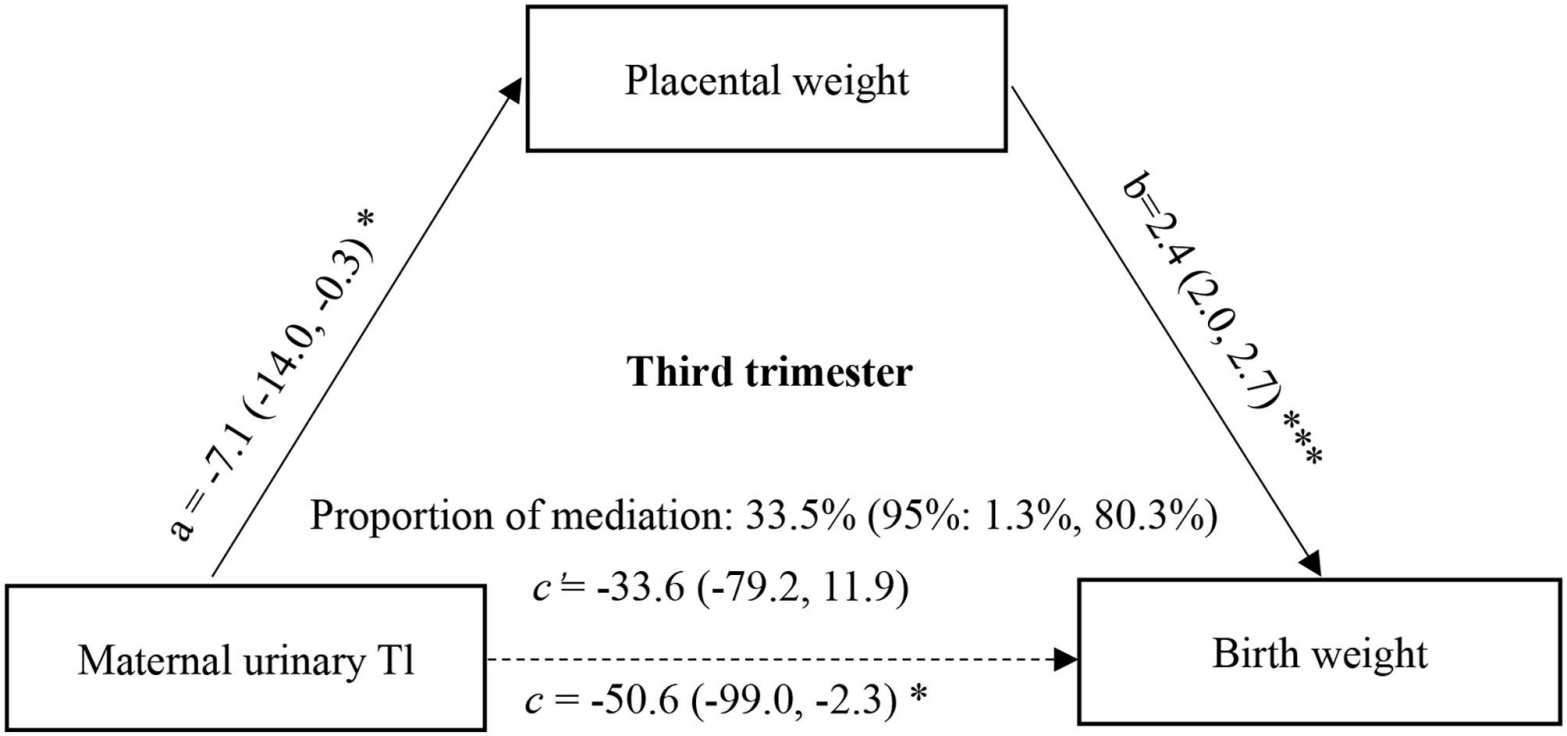
Mediation effect of placental weight on the association of maternal urinary Tl exposure during the third trimester with birth weight. a, c, and c' were estimated for the highest vs. the lowest tertile of ln-Tl exposure, and b was estimated for each unit increase in placental weight. All effect sizes were adjusted for final analyses: maternal age, maternal education, family income, gestational week, parity, gravidity, infant sex, vegetable consumption, fruit consumption. ^***^P < 0.001, ^*^P < 0.05.

Furthermore, to determine whether the change in placental weight is a potential mediator of the association between maternal urinary ln-Tl levels and birth weight, we performed the mediation analysis proposed by Mackinnon et al. with a series of multiple linear regression models after controlling for potential confounders (37). According to the theory of mediation analysis, four steps are needed to analyze the mediation effect. First, a significant relation (coefficient: c) of the independent variable (maternal urinary ln-Tl levels) to the dependent variable (birth weight) is required. Second, a significant relation (a) of the maternal urinary ln-Tl levels to the hypothesized mediating variable (placental weight) is required. Third, placental weight must be significantly related (b) to birth weight after maternal Tl exposure variable is introduced in the model. Fourth, the coefficient (c) relating the maternal urinary ln-Tl levels to birth weight must be larger (in absolute value) than the coefficient (c') relating the maternal urinary ln-Tl levels to birth weight in the regression model with both the maternal urinary ln-Tl levels and placental weight predicting birth weight. Then the indirect effect of the maternal urinary ln-Tl levels on the birth weight through the placental weight was evaluated as a × b, and the direct effect of the maternal urinary ln-Tl levels on birth weight was estimated as c'. A bootstrapping procedure using the Mplus was conducted using 5,000 resamples to test the mediation effect (38). In this approach, effects are assessed with bias corrected bootstrap CIs that are considered significant if the bias corrected 95% confidence intervals (95% CI) does not contain zero. The proportion of mediation was then calculated as 1 – (c'/c). This causal step approach to assessing mediation is the most widely used method to assess mediation. The directed acyclic graph (DAG) of mediation analysis can be seen in Figures 1 and 2.

Sensitivity analysis. To assess how robust were the associations to potential unmeasured confounding, we estimated the E-value and the limit of the confidence interval closest to the null (39). Briefly, this indicator represents the minimum strength of the association that one or more unmeasured confounder would need to have with both the exposure and the outcome to fully explain a specific exposure-outcome association, conditional on the measured covariates. The EValue package was used to calculate the E-value.

All the analyses were performed using R3.6.1 (R Development Core Team (40), https://www.r-project.org), and the “car,” “psych,” “multcomp,” “Hmisc,” “corrplot,” and “vcd” packages in R software were mainly used. All the tests were two-sided, and P <0.05 was considered to be statistically significant.

## Results

### General Characteristics of the Study Population

A total of 2,748 mother-newborn pairs were finally included in the present study. Table 1 summarizes the general characteristics of all the participants. The average neonate's birth weight and placental weight was 3193.4 g (*SD* = 406.9 g) and 521.3 g (*SD* = 52.1 g), respectively. Of the total participants, 1,926 (70.1%) were aged 30 years and over, 2,648 (96.4%) had term births (>37 gestational weeks), 1,485 (54.0%) had a male infant, 1,633 (59.5%) had 13 years of education or more, and 880 (32.0%) experienced passive exposure to smoke during pregnancy. The birth weight was significantly higher in pregnant women who had higher family income, gestational weeks >37, higher prepregnancy BMI, male infants, higher gravidity, and were primiparous.

**Table 1:** General characteristics of participants.

	**Number of participants** **n (%)**	**Birth weight (g)Mean ± SD**	***t*/F**	***P***
**Maternal age (years)**				
<25	73 (2.7)	3217.1 ± 426.7	1.74	0.157^a^
25–29	749 (27.3)	3166.8 ± 393.5		
30–34	1,060 (38.6)	3209.9 ± 401.1		
≥35	866 (31.5)	3194.2 ± 422.8		
**Maternal education (years)**				
≤ 12	1,115 (40.6)	3180.0 ± 417.8	1.67	0.189^a^
13-15	884 (32.2)	3192.0 ± 391.7		
>15	749 (27.3)	3215.0 ± 407.7		
**Maternal occupation**				
Housewife	259 (9.4)	3184.2 ± 452.2	0.57	0.682^a^
Unemployed	204 (7.4)	3191.5 ± 409.5		
Technician	540 (19.7)	3177.7 ± 387.6		
Service industry	1,475 (53.6)	3195.9 ± 405.1		
Others	270 (9.8)	3221.6 ± 410.0		
**Family income (×1000 Yuan)**				
<30	110 (4.0)	3073.6 ± 367.5	11.37	<0.001^a^
30	1,684 (61.3)	3171.9 ± 411.9		
100	821 (29.9)	3234.6 ± 395.7		
≥200	133 (4.8)	3310.9 ± 394.9		
**Passive smoking**				
No	1,868 (68.0)	3196.7 ± 407.1	0.62	0.536^b^
Yes	880 (32.0)	3186.4 ± 406.6		
**Paternal smoking**				
No	1,788 (65.1)	3187.6 ± 406.2	−1.03	0.303^b^
Yes	960 (34.9)	3204.3 ± 408.1		
**Physical activity (h/week)**				
<3.5	803 (29.2)	3183.7 ± 399.9	1.81	0.165*^*a*^*
3.5	1,008 (36.7)	3182.1 ± 397.8		
>3.5	937(34.1)	3213.9 ± 421.9		
**Tea consumption**				
No	2,480 (90.2)	3191.9 ± 404.8	−0.61	0.541^b^
Yes	268 (9.8)	3207.9 ± 426.6		
**Gestational week (Weeks)**				
<37	100 (3.6)	2549.5 ± 459.2	130.23	<0.001^a^
37–41	2,517 (91.6)	3206.9 ± 383.7		
≥42	131 (4.8)	3426.7 ± 334.0		
**Prepregnancy BMI (kg/m**^**2**^**)**				
Underweight (<18.5)	590 (21.5)	3095.1 ± 365.6	26.71	<0.001^a^
Normal (18.5–23.9)	1,766 (64.3)	3212.4 ± 404.9		
Overweight (≥24)	392 (14.3)	3255.9 ± 449.1		
**Gestational diabetes**				
No	2,110 (76.8)	3194.4 ± 401.0	0.24	0.812^b^
Yes	638 (23.2)	3190.1 ± 426.2		
**Infant sex**				
Male	1,485 (54.0)	3241.1 ± 402.8	6.72	<0.001^b^
Female	1,263 (46.0)	3137.3 ± 404.7		
**Parity**				
1	956 (34.8)	3156.6 ± 408.9	6.29	0.002^a^
2	1,664 (60.6)	3215.0 ± 405.8		
>2	128 (4.7)	3187.1 ± 389.3		
**Gravidity**				
1	749 (27.3)	3150.7 ± 395.6	5.08	0.002^a^
2	1,101 (40.1)	3200.6 ± 410.3		
3	597 (21.7)	3204.5 ± 396.5		
≥4	301 (11.0)	3251.5 ± 433.3		
**Meat consumption (times/week)**				
≤ 14	2,011 (73.2)	3199.9 ± 398.7	1.50	0.221^b^
>14	696 (25.3)	3177.1 ± 430.9		
Refused to answer or missing	41 (1.5)	3151.2 ± 381.2		
**Milk consumption (times/week)**				
<7	1,060 (38.6)	3199.2 ± 412.4	0.81	0.444^a^
7	1,520 (55.3)	3193.3 ± 403.5		
>7	110 (4.0)	3147.3 ± 408.8		
Refused to answer or missing	58 (2.1)	3180.2 ± 394.7		
**Fruit consumption (times/week)**				
<7	217 (7.9)	3170.3 ± 416.0	0.53	0.587^a^
7	2,274 (82.8)	3195.7 ± 406.8		
>7	207 (7.5)	3209.4 ± 400.4		
Refused to answer or missing	50 (1.8)	3125.0 ± 400.9		
**Vegetable consumption (times/week)**				
<14	208 (7.6)	3195.4 ± 396.6	0.02	0.981^a^
14	991 (36.1)	3191.1 ± 399.9		
>14	1,504 (54.7)	3194.1 ± 412.7		
Refused to answer or missing	45 (1.6)	3213.3 ± 422.0		

*BMI, body mass index*.

a *Statistics were estimated by ANOVA*.

b *Statistics were estimated by t-test*.

The median (25–75th) urinary Tl concentrations were 0.38 (0.23–0.60) μg/L in the first trimester and 0.25 (0.11–0.49) μg/L in the third trimester. After calibration with urinary creatinine and transformation by natural logarithm, the mean (±SD) ln-Tl exposure levels in the first and third trimester were 6.3 ± 0.6 ng/g creatinine and 6.1 ± 0.7 ng/g creatinine, respectively. The correlation analyses showed that the maternal Tl exposure levels during the first trimester were negatively associated with placental weight (*r* = −0.05, *P* = 0.016) and birth weight (*r* = −0.02, *P* = 0.466) and that placental weight was positively associated with birth weight (*r* = 0.38, *P* < 0.001) (Supplementary Figure 2). Similar results were observed among the maternal Tl exposure levels during the third trimester, placental weight, and birth weight (Supplementary Figure 3).

### Association Between Prenatal Tl Exposure and Birth Weight

Table 2 shows the unadjusted and adjusted associations of maternal exposure to Tl during pregnancy with birth weight. We observed a negative association between Tl exposure during the first trimester and birth weight after adjustment for potential covariates. Each IQR (0.72 ng/g creatinine) increment in the urine ln-Tl concentration was associated with a mean −13.1 g (95% CI: −32.7, 6.4 g) in birth weight. Besides, pregnant women with the second (β = −30.6 g, 95% CI: −70.4, 9.2 g) and third tertile (β = −42.7 g, 95% CI: −82.3, −3.1 g) ln-Tl concentrations in their urine had babies with lower birth weights than those with the first tertile of ln-Tl concentrations in their urine, and the trend analysis was statistically significant (*P* = 0.036).

**Table 2:** Associations of maternal exposure to Tl during pregnancy with birth weight and placental weight.

	**Birth weight (g)**		**Placental weight (g)**	
	**Crude β (95% CI)**	Adjusted β (95% CI)^*^	**Crude β (95% CI)**	Adjusted β (95% CI)^*^
**Ln-Tl concentrations (ng/g creatinine) measured in the first trimester (*****n*** **= 2,153)**				
Per IQR (0.72 ng/g creatinine) change	−8.1 (−29.1, 12.8)	−13.1 (−32.7, 6.4)	−3.2 (−5.9, −0.6)^*^	−3.2 (−5.8, −0.5)^*^
Tertile 1 (≤ 6.02)	1	1	1	1
Tertile 2 (6.02–6.48)	−38.4 (−81.0, 4.1)	−30.6 (−70.4, 9.2)	−6.1 (−11.5, −0.8)	−5.5 (−10.9, −0.1)^*^
Tertile 3 (≥6.48)	−25.2 (−67.5, 17.0)	−42.7 (−82.3, −3.1)^*^	−7.2 (−13.0, −2.4)	−8.1 (−13.4, −2.8)^**^
P for trend	0.246	0.036	0.005	0.003
**Ln-Tl concentrations (ng/g creatinine) measured in the third trimester (*****n*** **= 1,371)**				
Per IQR (0.76 ng/g creatinine) change	−29.5 (−51.4, −7.5)^**^	−21.1 (−42.4, 0.1)	−3.3 (−6.3, −0.2)^*^	−2.9 (−6.0, 0.1)
Tertile 1 (≤ 5.90)	1	1	1	1
Tertile 2 (5.90–6.38)	6.1 (−43.8, 56.0)	13.0 (−35.5, 61.6)	0.7 (−6.2, 7.5)	1.6 (−5.3, 8.5)
Tertile 3 (≥6.38)	−60.8 (−110.4, −11.2)^*^	−50.6 (−99.0, −2.3)^*^	−7.7 (−14.6, −0.9)^*^	−7.1 (−14.0, −0.3)^*^
P for trend	0.016	0.040	0.027	0.042

*CI, confidential interval*.

*Adjusted for final analyses: maternal age, maternal education, family income, gestational week, parity, gravidity, infant sex, vegetable consumption, and fruit consumption.

** *P < 0.01*,

* *P < 0.05*.

We also observed negative associations of Tl exposure during the third trimester with birth weight. After adjustment for potential confounders, every IQR (0.76 ng/g creatinine) increment in the urine ln-Tl concentration was associated with −21.1 g (95% CI: −42.4, 0.1 g) in birth weight. Compared with women with the first tertile ln-Tl concentrations in their urine, those with the third tertile ln-Tl concentrations in their urine had babies with significantly lower birth weights (β = −50.6 g, 95% CI: −99.0, −2.3 g), but those with the second tertile ln-Tl concentrations in their urine had babies with slightly higher birth weights (β = 13.0 g, 95% CI: −35.5, 61.6 g).

### Associations Between Maternal Exposure to Tl and Placental Weight

Table 2 shows the associations between maternal exposure to Tl and placental weight. We found significant negative associations of ln-Tl concentrations in both the first and third trimester with placental weight after adjustment for potential confounders. Each IQR (0.72 ng/g creatinine) increase in urine ln-Tl concentration during the first trimester was associated with −3.2 g (95% CI: −5.8, −0.5 g) in placental weight. Pregnant women with the third tertile of ln-Tl concentration exhibited a significant decrease (β = −8.1 g, 95% CI: −13.4, −2.8 g) in placental weight compared with those with the first tertile of ln-Tl concentration. A significant decreasing trend in placental weight was found among the three tertiles (*P* = 0.003). Every IQR (0.76 ng/g creatinine) increment in the urine ln-Tl concentration during the third trimester was linked to an average of −2.9 g (95% CI: −6.0, 0.1 g) in placental weight. We also observed a significantly lower birth weight in the third tertile of the urine ln-Tl concentration group compared with the first tertile of the urine ln-Tl concentration group (β = −7.1 g, 95% CI: −14.0, −0.3 g). A significant decreasing trend in placental weight was also observed among the three tertiles (*P* = 0.042). Besides, we found that placental weight increase was positively associated with birth weight (β = 2.5 g for each gram increase in placental weight, 95% CI: 2.3, 2.8 g).

### Mediation of Placental Weight Loss in the Association Between Maternal Tl Exposure During Pregnancy and Birth Weight

Since the association between maternal Tl exposure and birth weight was only significant at the highest vs. lowest tertiles of Tl exposure, the mediation effect of placental weight on the association between maternal Tl exposure and birth weight was estimated for the highest tertile of Tl exposure. The direct and indirect effects in the first trimester were −21.19 (95%CI: −57.85, 16.22) (*P* = 0.267) and −21.47 (95% CI: −36.01, −7.80) (*P* = 0.003), respectively. And the direct and indirect effects in the third trimester were −33.65 (95% CI: −78.50, 12.68) (*P* = 0.152) and −16.94 (95% CI: −34.07, −2.15) (*P* = 0.035), respectively. We found that 50.3% (95% CI: 15.979.2%) of the effects of Tl exposure in the first trimester on birth weight could be mediated by changes in placental weight (Figure 1), and 33.5% (95% CI: 1.3, 80.3%) of the effects of Tl exposure in the third trimester could be mediated by changes in placental weight (Figure 2).

Sensitivity analyses. The E-value for the adjusted associations were 1.43 for Tl exposure and birth weight, 1.57 for Tl exposure and placental weight, and 1.08 for placental weigh and birth weight in the first trimester. And the E-value for the third trimester is 1.50, 1.52, and 1.08, respectively (Supplementary Table 1).

## Discussion

In this study, we used a prospective birth cohort study to investigate the associations between maternal Tl exposure during pregnancy and birth weight and we further assessed the mediating role of placental weight change. We found that prenatal Tl exposure was negatively associated with birth weight and placental weight and that decreased placental weight may mediate the association between maternal Tl exposure and reduced birth weight. To the best of our knowledge, this is the first epidemiological study that provides evidence of the mediating role of placental weight change in the association between maternal Tl exposure and birth weight in China. Our study extended our understanding of the mechanism of reduced birth weight and provided insight for developing strategies to reduce exposure to environmental Tl to achieve better pregnancy outcomes.

Tl has been recognized as a highly toxic heavy metal (14, 16, 17). In humans, Tl is mainly excreted through feces and urine, with ~73% of the excretion occurring through urine (41, 42). Hence, we measured the concentration of Tl in urinary samples to analyze the Tl exposure levels among study participants (43). Tl was found in the urine of almost all the participants, suggesting that Tl is prevalent in the study population. Besides, the median Tl concentrations in our study were 0.38 and 0.25 μg/L in first and third trimesters, respectively, which are higher than those in the general population (geometric mean, 0.03 μg/L in Italy; median, 0.15–0.18 μg/L in the United States; arithmetic mean, 0.15 μg/L in Germany) and pregnant women (arithmetic mean, 0.18 μg/g creatinine in Spain; geometric mean, 0.17 μg/L in the United States) in developed countries (44–49). However, our findings are comparable to other studies conducted in China (22, 50–52). For example, a study conducted in Wuhan reported that the geometric mean value of maternal urinary thallium was 0.34, 0.36, and 0.34 μg/L for the first, second, and third trimesters, respectively (51). These data suggest that pregnant women in China are exposed to the potential risks of Tl. With rapid economic growth, the Tl emissions from anthropogenic activity have been rapidly increasing (53), thus more attention needs to be paid to the potential health effects of Tl exposure in pregnant women.

Research on the relationship between Tl and birth weight is limited. Several studies in developed countries suggested negative associations of maternal Tl exposure during pregnancy with birth weight (19, 20). For instance, Hoffman et al. observed a significant association between high-level exposure to Tl (urinary Tl >120 μg/L) during pregnancy and the risk of LBW (20). Govarts et al. found that cord blood Tl concentrations were negatively associated with birth weight in girls (19). Two studies conducted in China also found a significant association between maternal urinary Tl exposure during pregnancy and reduced birth weight (21, 22). For instance, a cross-sectional study reported a negative association between maternal and cord blood Tl concentrations with birth weight (21). A case-control study also observed that Tl concentrations measured in urine samples during pregnancy were positively associated with the risk of LBW among 816 pregnant women (22). In the present study, we also found a negative association between Tl exposure during pregnancy and birth weight. These studies consistently highlighted the adverse effects of prenatal exposure to Tl on fetal health. Therefore, pregnant women should be informed about the adverse effects of Tl. And the policy makers and clinical health workers should pay attention to Tl exposure in the environment and take corresponding control measures.

We further observed that maternal exposure to Tl was negatively associated with placental weight. While the association between maternal urinary Tl exposure and placental weight has rarely been examined, some animal experiments have indicated that exposure to other heavy metals, such as lead (Pb), can result in placental weight loss (54, 55), which may provide plausible mechanisms for the adverse effects of Tl. For example, Kaltenbach's study reported that an imbalance of oxidant and antioxidant activities in the placenta following Pb exposure contributed to placental damage (macroscopically observed as a decrease in placental weight) (56). A previous study indicated that maternal Tl exposure could trigger oxidative stress in the cell by increasing lipid oxidation (57), which may lead to placental damage. However, the mechanisms by which Tl affects the placenta remain unclear, and more studies are needed in the near future.

The results of mediation analyses suggested that the adverse effects of Tl exposure on birth weight could be mediated by placental weight change. This is the first study to analyze the mediating role of placental weight change in the association between prenatal Tl exposure and birth weight. However, there is a growing body of evidence that suggests that placental weight serves as an intermediary in the effect of prenatal factors on birth weight or fetal growth (28, 29, 52, 58–61). For example, previous studies suggested that placental weight mediated the association between maternal exercise during pregnancy and full-term low birth weight as well as preterm birth (59, 60). A cohort study found that the placenta mediated the relationship between prenatal air pollution exposure and birth weight (58). The recent study also reported that placental weight mediated the relationship between heavy metal cadmium exposure and birth weight (52, 61). These findings could provide more information for understanding the adverse effects of Tl exposure on fetal health.

The potential mechanisms of placental weight mediating the association between maternal exposure to Tl and birth weight are unclear. One possible mechanism is that Tl upregulates the mitochondrial RNA expression levels of placental inflammatory factors (TNF-a, IL-6, and CD68) by activating the NF-kB pathway, leading to local inflammation of the placenta (62). Another possible mechanism is the damage to placental mitochondrial function caused by oxidative stress and adenosine triphosphate (ATP) enzyme inhibition. Experimental studies suggested that Tl exposure could enhance intracellular reactive oxygen species production and decrease mitochondrial function by increasing levels of oxidative stress (57). A birth cohort study revealed a significant negative association between maternal Tl exposure and cord blood leukocyte mitochondrial DNA copy number (51). Additionally, because Tl^+^ and K^+^ have the same ionic radius and are difficult to distinguish in biofilms, Tl^+^ strongly competes with K^+^ of Na^+^/K^+^-ATPase even at low concentrations and causes swelling of isolated mitochondria, thus influencing placental mitochondrial function (63, 64). Mitochondria are primarily responsible for the generation of ATP and several other vital cellular functions (65, 66). Fetal growth is dependent on nutrient transportation across the placenta. These negative effects on the placenta can decrease placental nutrient and gas exchange, foster endocrine disruption, or lead to adverse birth outcomes, such as low birth weight (67).

One strength of our study is that we conducted a prospective birth cohort study, which can provide stronger evidence to illustrate the association between maternal urinary Tl exposure during pregnancy and reduced birth weight. Besides, face-to-face interviews and medical records allowed us to sufficiently adjust for potential confounding effects. This study also has some limitations. First, all the subjects were sampled from one hospital, which may limit the generalizability of our findings. In the next study, we will conduct a multicenter study to further confirm these findings. Second, we only measured placental weight, but outcomes may also be predicted by other physical parameters of the placenta, such as placental thickness, width, and length. Third, inorganic Tl includes monovalent Tl and trivalent Tl with different toxicities, but we only measured the concentration of total Tl in the urine samples. Fourth, we collected urine samples only in the first and third trimester but did not collect urine samples in the middle pregnancy. Therefore, this study can't estimate the associations of Tl exposure during middle pregnancy with placental weight and birth weight. Fifth, we did not collect information on variables such as fish consumption. As a result, we could not control for the confounding effects of these variables on our findings. Sixth, because the collection time of urine samples and birth outcomes is close in the third trimester, the ability of causal inference is relatively weak. Seventh, the E-value for the association of Tl exposure during the first and second trimester, placental weight and birth weight were ranged from 1.08 to 1.57. There is a moderate probability that unknown confounders could-but not necessarily would-explain away the observed associations. Eighth, mediation analysis results are correct as long as the assumed causal DAG depicts the true relationships. However, there are few studies on maternal urinary Tl exposure, birth weight or placenta weight. The causal associations among these three variables remain unclear. Therefore, more research works are needed on the role of placenta weight in the association between Tl exposure and birth weight. Ninth, the confidence intervals of mediation effects are relatively wide, which may be related to the small sample size.

## Conclusions

Prenatal Tl exposure was negatively associated with birth weight, and placental weight loss may mediate the adverse effects of Tl exposure on birth weight. These results could extend our understanding of the adverse effects of prenatal exposure to Tl on fetal health and suggest that pregnant women should take measures to reduce exposure to Tl during pregnancy, such as decreasing time spent in outdoor activities, consuming safe drinking water and food, and increasing their health awareness. Moreover, we call for more research to be conducted in the future to confirm our findings and analyze the mechanisms through which Tl adversely affects fetal health.

## Data Availability Statement

The datasets used and analyzed during the current study are available from the corresponding author on reasonable request.

## Ethics Statement

The studies involving human participants were reviewed and approved by the Ethics Committee of Guangdong Provincial Center for Disease Control and Prevention and was registered in the Chinese Clinical Trial Registry (ChiCTR-ROC-17013496). The patients/participants provided their written informed consent to participate in this study.

## Author Contributions

HZ and XS: Data curation, formal analysis, visualization, writing—original draft, writing—review and editing. YW: Data curation, formal analysis, methodology, project administration, validation. YY, JW, MD, DC, GC, LY, ZR, MZ, YL, and YP: Investigation. HC, QC, GH, XL, and JF: Methodology. JX: Project administration. JH: Software and Data curation. WZ: Data curation and investigation. QZ: Resources. LG: Project administration and resources. WM: Project administration. BZ: Conceptualization and funding acquisition, investigation, resources, and validation. TL: Conceptualization, funding acquisition, resources, supervision, writing—review and editing. All authors contributed to the article and approved the submitted version.

## Conflict of Interest

The authors declare that the research was conducted in the absence of any commercial or financial relationships that could be construed as a potential conflict of interest.
