# Supplementary material for: The Mediating Role of Placental Weight Change in the Association Between Prenatal Exposure to Thallium and Birth Weight: A Prospective Birth Cohort Study
Source: Front Public Health. 2021 Jul 2;9:679406. doi: 10.3389/fpubh.2021.679406 (PMC8283527; doi:10.3389/fpubh.2021.679406)
Supplement: Supplementary file 1 [file Data_Sheet_1.docx]

Supplementary material

**Figure of Contents**

**Table 1.** Adjusted β and 95% confidence intervals (95% CI) for the association between Tl during pregnancy, placental weight and birth weight (main analysis), and corresponding E-values.

**Figure 1.** The procedure of the Prenatal Environments and Offspring Health (PEOH) prospective birth cohort study.

**Figure 2.** The relationships among maternal urinary thallium levels in early pregnancy, placental weight, and birth weight.

**Figure 3.** The relationships among maternal urinary thallium levels in late pregnancy, placental weight, and birth weight.

**Table 1. Adjusted β and 95% confidence intervals (95% CI) for the association between Tl during pregnancy, placental weight and** **birth weight (main analysis), and corresponding E-values.**

| Exposure | Outcome | Adjusted β (95% CI) * | E-value | E-value (95% CI) |
| --- | --- | --- | --- | --- |
| **First trimester** |  |  |  |  |
| Tertile of ln-Tl | Birth weight (g) | -42.7 (-82.3, -3.1) ^a^ | 1.43 | 1.09, +inf |
|  | Placental weight (g) | -8.1 (-13.4, -2.8) ^a^ | 1.57 | 1.28, +inf |
| Placental weight (g) | Birth weight (g) | 2.6 (2.4, 2.9) ^b^ | 1.08 | 1.07, +inf |
| **Third trimester** |  |  |  |  |
| Tertile of ln-Tl | Birth weight (g) | -50.6 (-99.0, -2.3) ^a^ | 1.50 | 1.12, +inf |
|  | Placental weight (g) | -7.1 (-14.0, -0.3) ^b^ | 1.52 | 1.07, +inf |
| Placental weight (g) | Birth weight (g) | 2.4 (2.0, 2.7) ^b^ | 1.08 | 1.07, +inf |

* All effect sizes were adjusted for maternal age, maternal education, family income, gestational week, parity, gravidity, infant sex, vegetable consumption, and fruit consumption. ^a^ was estimated for the highest vs the lowest tertile of ln-Tl exposure, and ^b^ was estimated for each unit increase in placental weight.

First follow-up investigation during hospitalization for childbirth: A total of 4279 people completed the survey, and 1371 of them provided urine samples.

**The PEOH prospective birth cohort study**

Baseline investigation in the early pregnancy: A total of 4928 people completed the survey, and 2153 of them provided urine samples.

Early pregnancy

Middle pregnancy

Late pregnancy

Figure 1. The procedure of the Prenatal Environments and Offspring Health (PEOH) prospective birth cohort study.


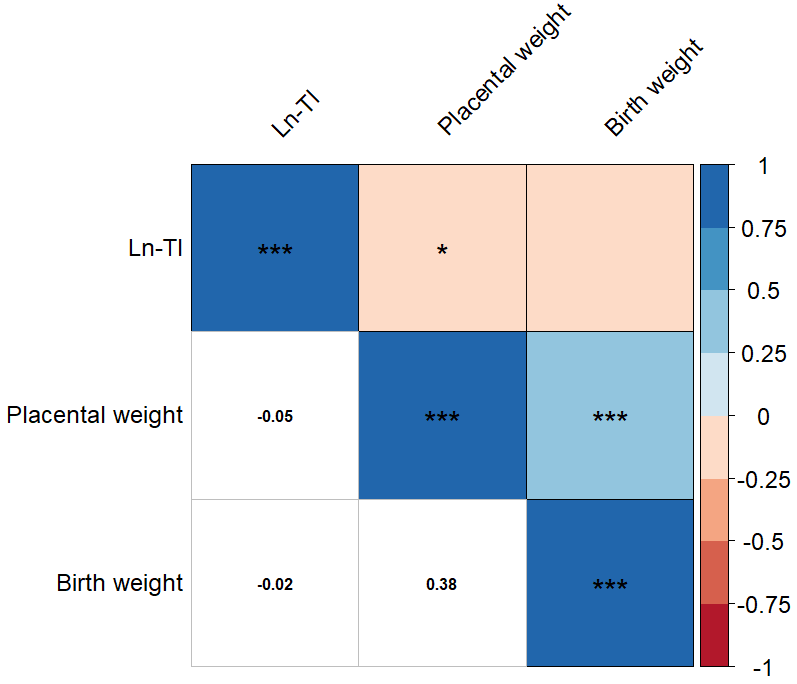


Figure 2. The relationships among maternal urinary thallium levels in early pregnancy, placental weight, and birth weight.


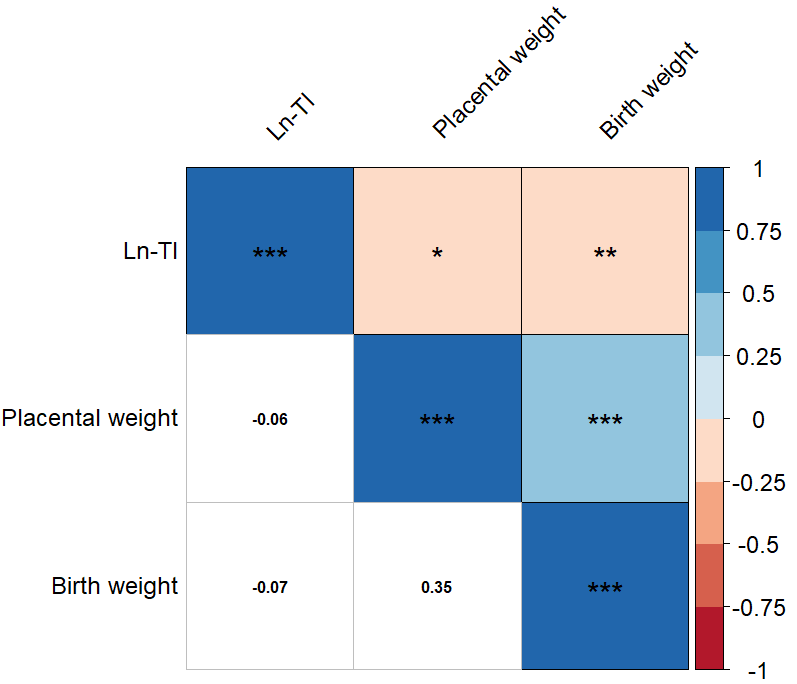


Figure 3. The relationships among maternal urinary thallium levels in late pregnancy, placental weight, and birth weight.
