# Supplementary material for: The Mediating Role of Placental Weight Change in the Association Between Prenatal Exposure to Thallium and Birth Weight: A Prospective Birth Cohort Study
Source: Front Public Health. 2021 Jul 2;9:679406. doi: 10.3389/fpubh.2021.679406 (PMC8283527; doi:10.3389/fpubh.2021.679406)
Supplement: Supplementary file 2 [file Data_Sheet_2.doc]

Table R1. Associations of maternal exposure to Tl during pregnancy with birth weight and placental weight.

|  | Birth weight (g) | |  | Placental weight (g) | |
| --- | --- | --- | --- | --- | --- |
|  | Crude β (95% CI) | Adjusted β (95% CI) ^*^ |  | Crude β (95% CI) | Adjusted β (95% CI) ^*^ |
| Ln-Tl concentrations (ng/g creatinine) measured in the first trimester (n=2153) |  |  |  |  |  |
| Per IQR (0.72 ng/g creatinine) change | -8.1 (-29.1, 12.8) | -13.1 (-32.7, 6.4) |  | -3.2 (-5.9, -0.6) * | -3.2 (-5.8, -0.5) * |
| Each unit increase in Ln-Tl concentrations (ng/g creatinine) | -11.3 (-40.4, 17.8) | -18.2 (-45.4, 8.9) |  | -4.5 (-8.1, -0.8) * | -4.4 (-8.1, -0.7) * |
| Ln-Tl concentrations (ng/g creatinine) measured in the third trimester (n=1371) |  |  |  |  |  |
| Per IQR (0.76 ng/g creatinine) change | -29.5 (-51.4, -7.5) ** | -21.1 (-42.4, 0.1) |  | -3.3 (-6.3, -0.2) * | -2.9 (-6.0, 0.1) |
| Each unit increase in Ln-Tl concentrations (ng/g creatinine) | -38.8 (-67.6, -10.0) ** | -27.8 (-55.8, 0.1) |  | -4.3 (-8.3, -0.3) * | -3.9 (-7.9, 0.1) |

CI, confidential interval

^*^ Adjusted for final analyses: maternal age, maternal education, family income, gestational week, parity, gravidity, infant sex, vegetable consumption, and fruit consumption.

***P < 0.001, **P < 0.01, *P < 0.05.
